# Surgical treatment of distal tibia non-unions with a retrograde intramedullary nail as a therapy option to prevent amputation

**DOI:** 10.1038/s41598-025-00318-6

**Published:** 2025-05-07

**Authors:** Sebastian Findeisen, Jessica Böpple, Michael Tanner, Tim Bewersdorf, Christian Schamberger, Thomas Ferbert, Tobias Grossner, Gerhard Schmidmaier, Matthias Miska

**Affiliations:** Clinic for Trauma and Reconstructive Surgery, Center for Orthopedics, Trauma Surgery and Paraplegiology, University Hospital Heidelberg, Schlierbacher Landstraße 200a, 69118 Heidelberg, Germany

**Keywords:** Non-union therapy, Hindfoot arthrodesis, HAN, Clinical trials, Risk factors

## Abstract

Compared to other long bones, non-unions of the tibia after fractures are more likely. Due to surgical debridement, the residual bone stock in the distal part of the bone may be insufficient for adequate fixation of an implant. Thus, treatment with a retrograde nail with the additional performance of an arthrodesis of the hindfoot may be an alternative. The aim of this study was to examine the consolidation and reinfection rates of non-unions of the distal tibia treated with a retrograde arthrodesis nail. A total of 27 patients between 2010 and 2018 were included according to our inclusion criteria. The modified Lane Sandhu Score was used for the radiographic evaluation. The osseous consolidation was validated after 1 year as well as after 2 years. Furthermore, we investigated differences in surgical treatment and treatment-associated complications. One year after the initial therapy 48% of the patients showed osseous consolidation, 75% showed consolidation after two years. 14% underwent amputation later. The majority of the patients (77%) received the ETN PROtect (Expert Tibia Nail PROtect) retrogradely, while the rest were obtained with the HAN (Hindfoot Arthrodesis Nail). Major revision surgeries were required in 26% of patients, while minor revisions were necessary in 15% of patients. In conclusion, treatment with a retrograde nail is a safe treatment option for distal tibia non-unions with a satisfactory overall fusion rate. Therefore, it can be an effective option especially in cases, where the fixation of the implant is difficult, in order to prevent amputation.

## Introduction

The rate of patients ending up with a non-union after fractures of the tibia is higher than compared to other long bones^1^. The prevalence is between 1 and 10% of the patients^1^, mainly located in the distal fifth of the tibia. Patients who have sustained open and/or highly comminuted fractures are predisposed to a high risk of infected non-unions. This is due to the destruction of bone substance and blood supply, persistent low-grade infections, and the potential for posttraumatic arthritis partly associated with infection^4^.

Due to the extensive surgical debridement required, in some cases the residual bone stock in the distal part of the bone may be insufficient for adequate fixation of an implant. Therefore, in some cases the ankle joint needs to be sacrificed to achieve sufficient stability for example using a locking plate, even in cases with mainly intact joint surface. An alternative treatment option is hindfoot arthrodesis, a procedure which is more commonly employed in cases with post-traumatic osteoarthritis, chronic instabilities, or deformities of the upper and/or the lower ankle joint^2–4^. The objective of this procedure is to achieve bony fusion of the calcaneus, talus and distal tibia, thereby reducing existing pain and facilitating full weight-bearing of the affected limb.

Numerous studies have demonstrated that these results can be attained with hindfoot arthrodesis one year following surgery^2,3^. According to current scientific data, the fusion rates for hindfoot arthrodesis are acceptable^3^.

The HAN (Expert Hindfoot Arthrodesis Nail, DePuy Synthes) is currently used most frequently for this purpose. It has been specially designed to improve tibiotalocalcaneal arthrodesis in cases of severe foot and ankle deformities, osteoarthritis, instabilities and skeletal defects^4^. Since it is only available in a maximum length of 240 mm^5^, it may not be suitable for long bone defects of the distal tibia, as it does not offer sufficient proximal anchoring options.

Another intramedullary nail, which is primarily used for fractures of the tibial shaft, metaphysis and certain intra-articular fractures of the tibial head, is the ETN PROtect (Expert Tibia Nail PROtect, DePuy Synthes)^6^. It may be used off label for arthrodesis of the hindfoot in a retrograde configuration and especially in revision cases. Additionally, it offers antibiotic properties due to its Gentamycin coating and is available in larger lengths than the HAN^6,7^.

The aim of this study was to collect additional outcome data of non-unions of the distal tibia treated with a retrograde arthrodesis nail regarding the consolidation and reinfection rate, as the available data is limited. The primary endpoint is radiological confirmation of osseous consolidation one year postoperatively, in addition to the absence of infection.

## Materials and methods

### Patients

Between May 2010 and March 2018, a total of 27 patients with non-union of the distal tibia undergoing treatment with a retrograde arthrodesis-nail, were included. The rationale for this intervention was the presence of inadequate bone stock, measuring less than 2,5 cm between the non-union and the articular surface of the upper ankle joint.

Inclusion criteria were an operative treatment in our department on account of a non-union with limited bone stock of the distal tibia, a minimum age of 18 years and written informed consent. Exclusion criteria were a follow-up period of less than one year, a lack of informed consent as well as an age of less than 18 years. Both, infected as well as aseptic non-unions were included.

This study was conducted in accordance with the current declaration of Helsinki and was approved by the local Ethics Committee (S-033/2014). All patients agreed with the study protocol and gave their written informed consent.

Out of 27 patients who met the inclusion criteria, five patients had to be excluded: all of them were lost to follow-up.

Five patients received a HAN, whereas in seventeen patients the retrograde ETN Protect was used. The decision regarding the selected nail was based on the following criteria: prevalence of infection/osteitis, defect size, the feasibility of adequate stabilisation with the shorter HAN, and the necessity for revisions. In this case, the ETN was found to be the optimal choice. The Masquelet induced membrane technique was utilized in cases of confirmed infection, atrophic avascular non-unions, and segmental bone defects. All patients had a relative indication for amputation of the affected limb due to pre-existing infections, critical soft tissue conditions, or extensive bone defects.

### Bone grafts, bone substitutes and growth factors

The different treatment options for all patients were consistent with previously published data from our department. In this study a variety of bone grafts and substitutes were used to fill the resulting bony defect in the distal tibia. As bone substitute tricalcium phosphate (Vitoss®, Mahwah, NJ, USA) was used. In some cases, no bone substitute was employed, whereas in others, a combination of autologous bone and Vitoss® was utilized. Furthermore, in accordance with the previously published departmental guidelines, some patients received additional growth factors, such as rhBMP-2 or rhBMP-7.

Due to the long recruitment period and the accumulation of knowledge over time, the combination was determined individually by the surgeon, with consideration given to availability, resulting in a high degree of variance overall.

### Radiographic evaluation

**Fig. 1:**
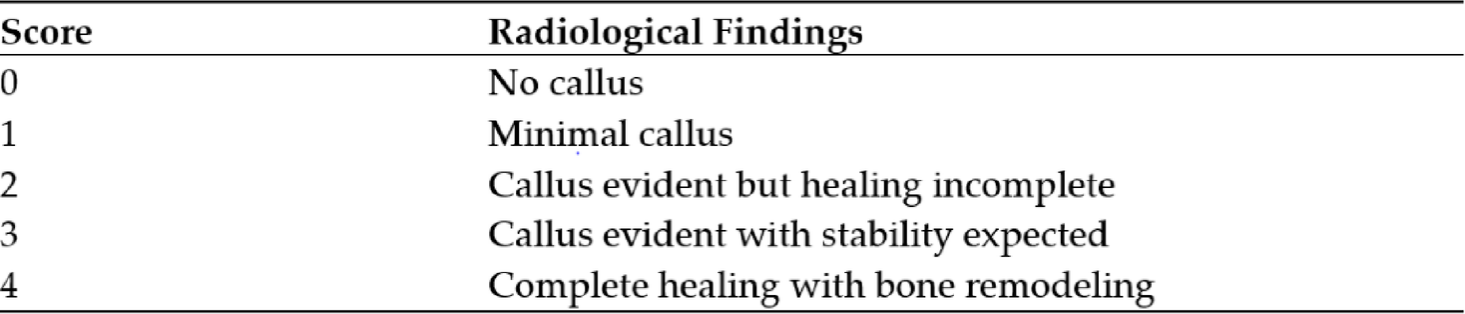
Modified Lane–Sandhu-score^8^.

The Lane Sandhu Score with its radiological criteria was used for the radiographic evaluation of the osseus consolidation. A score $\le 2$ is considered as unstable and not consolidated while a score $\ge 3$ is considered to be stable and consolidated (Fig. 1). The radiographic images were assessed after a minimum of 1 year and after2 years.

### Complications

The number of persistent non-unions, as well as amputations, was analysed. In conjunction with this, the number of major and minor revision surgeries was also examined. Major revision surgery is defined as surgery that necessitates bony revision, for example due to persistent non-union, or where there is a necessity for modification of the nail. Minor revisions are limited to soft tissue revision surgery, for example due to persistent wound secretion.

### Data analysis

Statistical analysis was performed using IBM SPSS Statistics 29.0.1.1. as well as Stata statistical software (version 18.0, StataCorp, United States). Given the inhomogeneity of the collective, a comparison of the osteosynthesis groups was not meaningful. Thus, descriptive statistics were performed, with the exception of a few values, including age, gender, smoking status, diabetes, and BMI. The assumption was made that the data were not normally distributed due to the relatively small sample size of each group. Consequently, the Wilcoxon signed-rank test was utilized to facilitate a comparison between the two groups for the specified parameters. The analysis of categorical data was performed using the Fisher exact test. A p-value of less than 0.05 was deemed to be statistically significant.

## Results

### Patient characteristics

Initially, 27 patients were included in the study. Of these, 5 were lost to follow-up. Of the remaining 22 subjects, 20 patients were male (91%). The median age was 52 years and the median BMI was 27 kg/m^2^. 8 of the 22 patients were smokers and 7 had diabetes. The ASA scores are shown in Table 1 as well as all the above-mentioned characteristics.

**Table 1:** Demographic data of the included patients.

n	22
Age	
Median (IQR) [years]	52 (45–60)
Gender	
Male	20 (91%)
Female	2 (9%)
BMI	
Median (IQR) [kg/m^2^]	27 (25–30)
Smoker	
No	14 64%)
Yes	8 (36%)
Diabetes	
No	15 (68%)
Yes	7 (32%)
ASA score	
1	3 (14%)
2	9 (41%)
3	10 (45%)
Residual bone stock distal of the tibia non-union	
Median (IQR) [cm]	1,7 (1,2–2,2)
Initial fracture type	
Open	14 (64%)
Closed	8 (36%)
Type of non-union	
Aseptic	16 (73%)
Infected	6 (27%)

Regarding the initial fractures, 10 (45%) out of the 22 patients had fractures of the tibia, the remaining 12 (55%) patients had fractures of the tibia and the fibula. Moreover, 2 (9%) were distal diaphyseal and resulted in large diametaphyseal defect after osteitis and previous treatments, while the other 91% were metaphyseal. In total, 14 patients (64%) had an open fracture whereas the remaining 8 patients (36%) had closed fractures.

### Surgical treatment

**Fig. 2:**
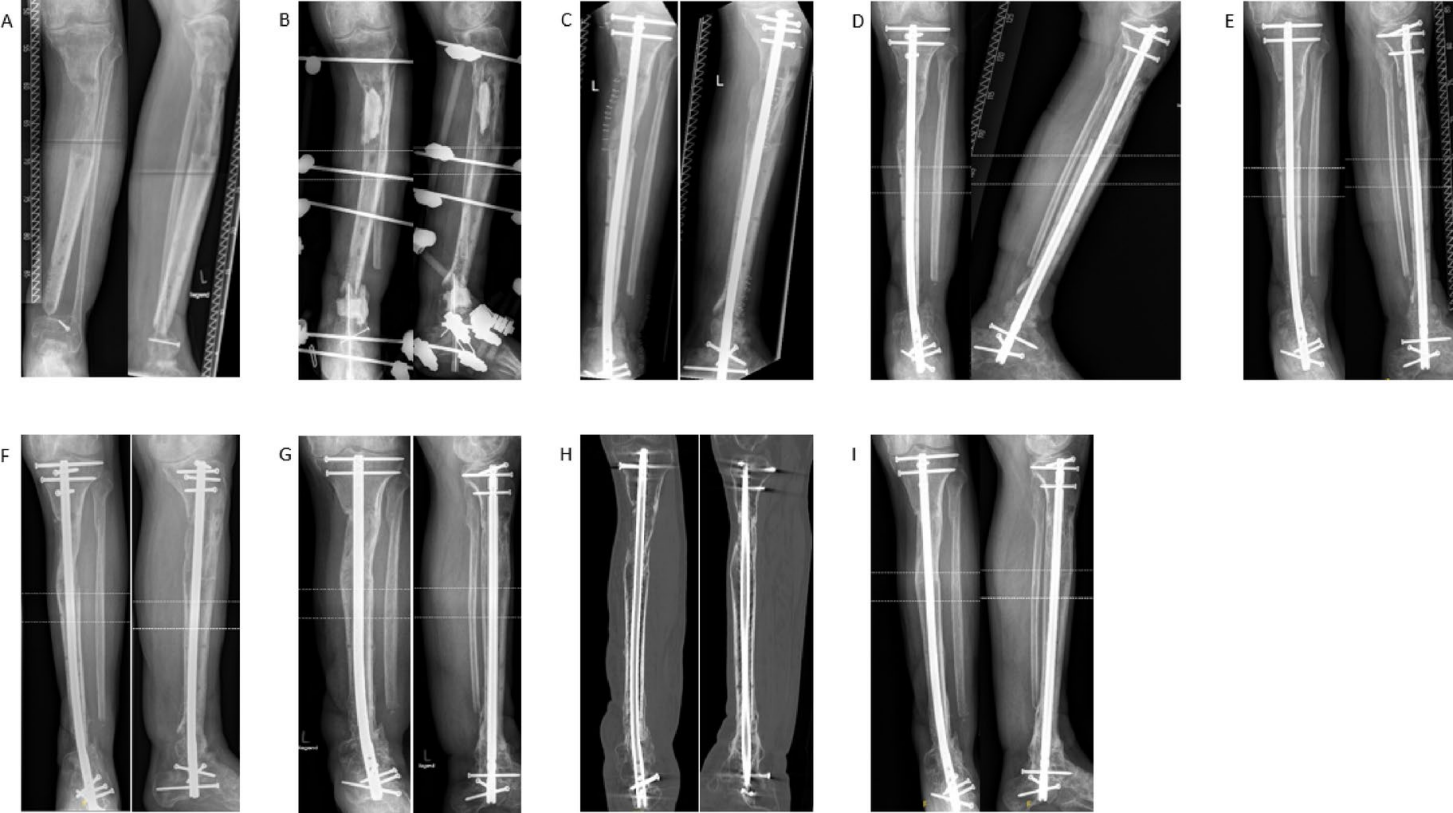
Example of a patient with a distal tibia non-union undergoing a two-step procedure receiving a ETN protect (**A**) initial X-rays presenting the first time in our department (**B**) X-rays two days after the first step of the Masquelet therapy with debridement of the non-union and resection of the sclerotic avascular bone, the bone defect was filled with a PMMA spacer, stabilization was achieved using an external fixator (**C**) X-ray two days postoperative after Step 2 of the induced membrane technique with change to an ETN protect, removal of the spacer and augmentation of the bony defect with autologous bone in RIA technique from the ipsilateral femur in combination with VITOSS and BMP-7 (**D**) X-ray image 6 weeks postoperative (**E**) X-ray image 12 weeks postoperative (**F**) X-ray image 6 months postoperative (**G**) X-ray image 1 year postoperative (**H**) CT scan 1 year postoperative with complete bony consolidation (**I**) X-ray image 2 years postoperative with ongoing remodelling.

The majority of the patients (n = 17, 77%) received an ETN PROtect as intramedullary nail, while five patients treated with a HAN. In 13 patients (59%) RIA from the contralateral femur was used as bone graft technique. 3 patients (14%) received ABG from RIA and the iliac crest. In one patient (5%) only the iliac crest was used, in one (5%) RIA and the distal fibula were used in combination. Four patients (18%) did not receive any bone graft. Furthermore, in 14 patients (64%) Vitoss was combined with the growth factor BMP-7 in 2 patients (9%) Vitoss with BMP-2, while six patients (27%) did not receive a bone substitute or growth factor. A clinical case is exemplarily shown in Fig. 2

### Consolidation and complications

One-year post-op, 48% of the patients showed osseous consolidation, while 77%showed consolidation after two years. 5 patients (23%) did not. Out of these 5, 3 (14%of all patients) were amputated.

## patients (9%) were maintained with a dynamization of their implants during healing process.

Revision data is illustrated in Table 2.

**Table 2:** Presentation of data on major and minor revisions.

n	22
Major revisions	
None	15 (68%)
One	3 (14%)
More	4 (18%)
Minor revisions	
None	18 (82%)
One	3 (14%)
More	1 (5%)
Revisions over all	
No	11 (50%)
Yes	11 (50%)

Regarding consolidation, there is no significant correlation regarding the smoking status (p = 1,00), diabetes (p = 1,00) or fracture type (p = 1,00). There is no statistic significant correlation regarding the reinfections within the consolidated group and those who did not consolidate (p = 0,1). The exact numbers are shown in Table 3.

**Table 3:** Analysing consolidation based on risk factors.

	Non-consolidated	Consolidated	*p*-Value
n	5	17	
Smoking			1,00
Yes	2 (40%)	6 (35%)	
No	3 (60%)	11 (65%)	
Diabetes			1,00
Yes	2 (40%)	5 (29%)	
No	3 (60%)	12 (71%)	
Fractures			1,00
Open	3 (60%)	11 (65%)	
Closed	2 (40%)	6 (35%)	
Reinfection			0,10
Yes	3 (60%)	3 (18%)	
No	2 (40%)	14 (82%)	

Furthermore, data regarding the consolidation as well as the revision surgeries required in infected and aseptic non-unions were analysed. The results of this analysis are presented in Table 4.

**Table 4:** Comparison of data based on the breakdown of infected and aseptic non-unions.

	Aseptic non-unions	Infected non-unions	*p*-Value
N	16	6	
Consolidation after 1 year			1,00
No	9 (56%)	3 (50%)	
Yes	7 (44%)	3 (50%)	
Consolidation in general			1,00
No	4 (25%)	1 (17%)	
Yes	12 (75%)	5 (83%)	
Major revision			0,50
No	12 (75%)	3 (50%)	
One	2 (12%)	1 (17%)	
More	2 (12%)	2 (33%)	
Minor revision			0,66
No	12 (75%)	6 (100%)	
One	3 (19%)	0 (0%)	
More	1 (6%)	0 (0%)	

## Discussion

The aim of this study consisted of an in-depth analysis of the characteristics and outcomes of patients treated with a retrograde arthrodesis nail as a treatment option for distal tibia non-unions. The most relevant finding of our study is that for patients with distal tibial non-unions, where insufficient bone stock remains for the fixation of a standard osteosynthesis following surgical debridement, with the use of a retrograde arthrodesis nail represents a viable alternative therapeutic option, demonstrating equivalent consolidation rates compared to other treatment modalities.

Some studies already have investigated treatment options for distal tibia non-unions. Reed et al.^9^ for example investigated the use of blade plates for distal tibia metaphyseal non-unions as an adequate therapy with successful results. Other treatment options that have been explored include the use of an intramedullary nail, provided there is sufficient bone stock, or an external fixator, such as the Ilizarov fixator^10–14^. Eralp et al.^13^ and Cunningham et al.^12^ demonstrated positive outcomes with various distraction techniques for the treatment of distal tibia non-unions or when using the Ilizarov fixator. Cunningham et al.’s study of 13 patients revealed a higher consolidation rate; however, the complication rate also appeared to be elevated, with three patients requiring plastic surgery^12^. Eralp et al. reported a 92.6% consolidation rate with the Ilizarov fixator, though all of their patients had aseptic non-unions^13^. However, few focused on treatment with a retrograde nail, leading to a paucity of data. Mosca et al.^15^ for instance analysed the data of 8 patients with aseptic distal tibial non-unions treated with a retrograde intramedullary nail. In their study, all 8 patients showed osseous consolidation after an average time of 22.8 weeks. Good results were also shown in terms of pain and return to work. However, Mosca et al.^15^ only investigated aseptic non-unions, which could explain the higher consolidation rate compared to our data. Ebraheim et al.^16^ also investigated possible treatment options for distal tibial non-unions. However, they did not limit themselves to one treatment option. The overall consolidation rate in their study was 88%. Compared to our patients, an intramedullary nail was used when the distal bone stock was sufficient, i.e. when the non-union was more diaphyseal. This showed the lowest revision rate. Ebraheim et al.^16^ predominantly used plate osteosynthesis for metaphyseal pseudarthrosis and an external fixator in the presence of severe infection. As a result, the data are only comparable to a limited extent. Another study dealing with distal but also tibial shaft non-unions is the study by Zhao et al.^17^. Here, a consolidation rate of 100% was shown in 12 patients. In comparison to our group, however, a locking plate could be used due to sufficient bone stock.

In general, smoking and diabetes are identified as risk factors for osseous consolidation^18–20^. Looking at our results regarding consolidation in smokers and diabetics, there are no significant differences regarding consolidation in our group. A review of literature reveals a lack of information regarding the influence of smoking and diabetes on consolidation in hindfoot arthrodesis. In some cases, patients with diabetes were excluded from study populations based on the pre-established inclusion criteria.^21^. However, in other instances, the potential increased risk of infection was noted^20,22^.

The present study reveals a mild tendency with regard to the reinfection rate and influenced bone healing (reinfection rate of 60% vs. 18% of those who did not consolidate vs. those who consolidated respectively). The lack of statistical significance may be attributed to the relatively small sample size. This finding is consistent with the current state of the literature^16,20^.

Moreover, looking at the infection rate six patients had a reinfection afterwards, which corresponds to 27% of the collective. In comparison to the findings of Zhao et al.^14^, who were unable to identify any instances of infection in their 12 patients, our data indicates an elevated infection rate. It should be noted, however, that the cohort of Zhao et al.^14^ is smaller and includes fewer patients with infectious non-unions. Mosca et al.^15^ similarly reported no postoperative infection, but did note the occurrence of soft tissue irritation in three patients and prolonged wound healing in one patient. All patients presented with aseptic non-unions. In summary, the reinfections observed in our collective may be attributed to the inclusion of infected non-unions.

Regarding nails implanted, ETN PROtect was employed more often. Haubruck et al.^23^, published an article mentioning that ETN PROtect reduces implant-associated infections and osteomyelitis not only in general but also in initially contaminated fractures. This matches the results of Moghaddam et al.^7^ and Franz et al.^24^. In general, intramedullary nails demonstrate favourable outcomes in the management of tibial fractures^25,26^. This may be attributed to the minimally invasive approach, which may be associated with a reduced incidence of infection^27,28^. Furthermore, early full weight-bearing of the affected limb can also facilitate bone consolidation.

Our study showed a rate of 14% (3 patients) necessitating amputation due to persistent infection, in some cases with a septic course. There is very limited data on amputation rates in hindfoot arthrodesis for septic non-unions of the distal tibia. Ebraheim et al.^16^, had one amputation due to persistent non-union. This corresponds to about 3%. However, it should be mentioned that different treatment options were used in the study, which is why the data are only comparable to a limited extent. Furthermore, there are some studies regarding the amputation rate after hindfoot arthrodesis in general. The study by Macknet et al.^29^ showed an amputation rate of 20%. In this case, the primary indication for amputation was also persistent infection and non-union. In contrast, the study by Ersin et al.^30^ showed an amputation rate of 0%. However, in his study he only included patients with Charcot neuropathy without infections or open fractures. Compared to this, Wukich et al.^31^ report a 3.2–5.3% amputation rate in their study in a total of 117 patients who had received arthrodesis of the ankle and hindfoot. Most of their patients had Charcot neuropathy, but some also had osteoarthritis and revision arthrodesis as indications. The patients included in our study had, in contrast to the studies mentioned above, a distinctly different indication for the use of a hindfoot nail, for instance, painful post-traumatic osteoarthritis following ankle joint injuries. In our patients, however, treatment with a retrograde nail is often the last treatment option before performing an amputation due to chronic osteitis or severe bone defects which do not provide a sufficient anchoring option in the bone, making hindfoot arthrodesis with bone grafting the last reasonable treatment option. This may justify a slightly higher amputation rate. Nonetheless, the incidence of lower limb amputations remains low, supporting the high prevalence of osseous consolidations.

It is important to note that our study was subject to certain limitations. Firstly, the results cannot be generalised to a larger population due to the limited number of patients included in the trial. Secondly, as this was a retrospective study, the results may be less significant than those from a prospective study. To confirm the efficacy of hindfoot arthrodesis as a treatment option for distal tibia non-unions, further well-powered randomized controlled trials in a larger group are required.

## Conclusion

In patients with distal tibial non-union where adequate stability cannot be achieved using standard implants due to insufficient bone stock and where amputation is already considered, hindfoot arthrodesis with a retrograde intramedullary nail represents a viable therapeutic option with a satisfactory overall bony fusion rate. It provides the requisite stability and the potential for dynamization, which may positively influence bone healing. In order to achieve consolidation, infection control and adequate treatment of the bone defect are of course essential. For this purpose, the Masquelet technique represents a satisfactory treatment concept.

## Data Availability

The datasets used and/or analysed during the current study are available from the corresponding author on reasonable request.
